# *QuickStats:* Infant Mortality Rate, by Urbanization Level* — National Vital Statistics System, United States, 2007 and 2015

**DOI:** 10.15585/mmwr.mm6641a8

**Published:** 2017-10-20

**Figure:**
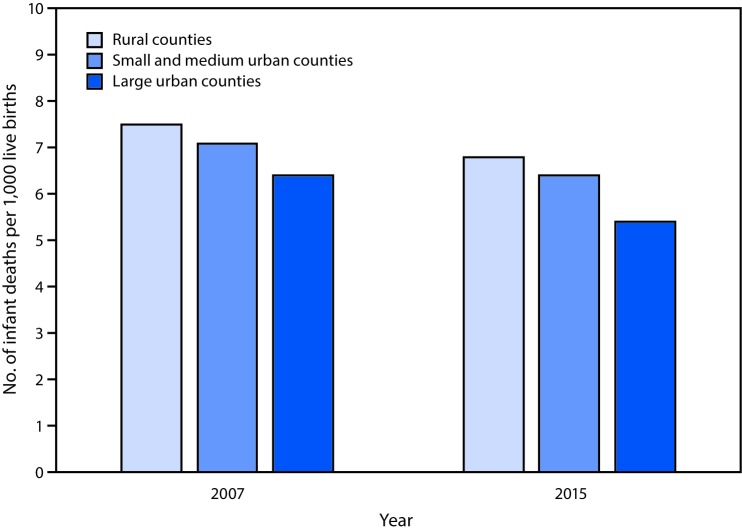
In both 2007 and 2015, infant mortality rates were highest in rural counties (7.5 infant deaths per 1,000 live births and 6.8, respectively). Rates were lower in small and medium urban counties (7.1 in 2007 and 6.4 in 2015) and lowest in large urban counties (6.4 in 2007 and 5.4 in 2015). For all three urbanization levels, infant mortality rates were significantly lower in 2015, compared with rates in 2007.

**Source:** National Vital Statistics System, linked birth/infant death period files, 2007 and 2015. https://www.cdc.gov/nchs/nvss/linked-birth.htm.

